# Impaired Fear Extinction Due to a Deficit in Ca^2+^ Influx Through L-Type Voltage-Gated Ca^2+^ Channels in Mice Deficient for Tenascin-C

**DOI:** 10.3389/fnint.2017.00016

**Published:** 2017-08-02

**Authors:** Fabio Morellini, Aleksey Malyshev, Maxim Volgushev, Marina Chistiakova, Giorgi Papashvili, Laetitia Fellini, Ralf Kleene, Melitta Schachner, Alexander Dityatev

**Affiliations:** ^1^Institute for Biosynthesis of Neural Structures, Zentrum für Molekulare Neurobiologie Hamburg, Universitätsklinikum Hamburg-Eppendorf Hamburg, Germany; ^2^Research Group Behavioral Biology, Zentrum für Molekulare Neurobiologie Hamburg, Universitätsklinikum Hamburg-Eppendorf Hamburg, Germany; ^3^Department of Neurophysiology, Ruhr-University Bochum Bochum, Germany; ^4^Institute of Higher Nervous Activity and Neurophysiology, Russian Academy of Sciences Moscow, Russia; ^5^Department of Psychological Sciences, University of Connecticut Storrs, CT, United States; ^6^Center for Neuroscience, Shantou University Medical College Shantou, China; ^7^Keck Center for Collaborative Neuroscience and Department of Cell Biology and Neuroscience, Rutgers University Piscataway, NJ, United States; ^8^Molecular Neuroplasticity Group, German Center for Neurodegenerative Diseases (DZNE) Magdeburg, Germany; ^9^Medical Faculty, Otto-von-Guericke University Magdeburg, Germany; ^10^Center for Behavioral Brain Sciences (CBBS) Magdeburg, Germany

**Keywords:** extracellular matrix, synaptic plasticity, learning, fear conditioning, L-type Ca^2+^ channels, extinction

## Abstract

Mice deficient in the extracellular matrix glycoprotein tenascin-C (TNC^−/−^) express a deficit in specific forms of hippocampal synaptic plasticity, which involve the L-type voltage-gated Ca^2+^ channels (L-VGCCs). The mechanisms underlying this deficit and its functional implications for learning and memory have not been investigated. In line with previous findings, we report on impairment in theta-burst stimulation (TBS)-induced long-term potentiation (LTP) in TNC^−/−^ mice in the CA1 hippocampal region and its rescue by the L-VGCC activator Bay K-8644. We further found that the overall pattern of L-VGCC expression in the hippocampus in TNC^−/−^ mice was normal, but Western blot analysis results uncovered upregulated expression of the Ca_v_1.2 and Ca_v_1.3 α-subunits of L-VGCCs. However, these L-VGCCs were not fully functional in TNC^−/−^ mice, as demonstrated by Ca^2+^ imaging, which revealed a reduction of nifedipine-sensitive Ca^2+^ transients in CA1 pyramidal neurons. TNC^−/−^ mice showed normal learning and memory in the contextual fear conditioning paradigm but impaired extinction of conditioned fear responses. Systemic injection of the L-VGCC blockers nifedipine and diltiazem into wild-type mice mimicked the impairment of fear extinction observed in TNC^−/−^ mice. The deficiency in TNC^−/−^ mice substantially occluded the effects of these drugs. Our results suggest that TNC-mediated modulation of L-VGCC activity is essential for fear extinction.

## Introduction

Tenascin-C (TNC) is prominently expressed in various tissues during development. In the developing central nervous system, TNC is involved in regulating the proliferation of oligodendrocyte precursor cells and astrocytes. TNC expression is downregulated in the adult brain, except for the areas that maintain neurogenesis into adulthood, such as the hippocampus and hypothalamus (Wiese et al., 2012). After injury, TNC expression is upregulated in neurons responding to the insult. TNC supports spinal cord regeneration by promoting axonal regrowth and synapse formation in the spinal cord caudal to the lesion site (Yu et al., 2011).

Furthermore, TNC plays an important role in adult hippocampal plasticity (reviewed by Senkov et al., 2014). Interestingly, only specific forms of synaptic plasticity induced by protocols that involve activation of L-type voltage-gated Ca^2+^ channels (L-VGCCs), such as repeated theta-burst stimulation (TBS) of Schaffer collaterals, application of the K^+^ channel blocker tetraethylammonium, or low-frequency stimulation-induced long-term depression, were impaired in constitutively TNC deficient (TNC^−/−^) mice (Evers et al., 2002). Moreover, reduction in the magnitude of TBS-induced long-term potentiation (LTP) in TNC^−/−^ mice was occluded by a decrease in LTP in the presence of nifedipine, a blocker of L-VGCCs. Nifedipine did not affect LTP in TNC^−/−^ mice but reduced LTP in wild-type mice to the levels seen in the mutants, supporting the view of a link between TNC and L-VGCCs (Evers et al., 2002). A follow-up study revealed elevated power of hippocampal and cortical theta and gamma oscillations in TNC^−/−^ mice (Gurevicius et al., 2009). The increase of the gamma power was specific to the CA1 region. In the dentate gyrus, the gamma power was not changed. Furthermore, the observed changes in synaptic transmission and LTP of TNC^−/−^ mice were specific for CA3-CA1 connections and not found in the dentate gyrus at perforant path synapses. At the behavioral level, TNC^−/−^ mice showed increased exploratory activity in a novel environment, decreased anxiety and delayed adaptation to daylight reversal (Morellini and Schachner, 2006).

Selective impairment of LTP induced by the protocols that involve L-VGCC activation in TNC^−/−^ mice suggests that TNC deficiency leads to impairment of the expression and/or functionality of these channels, which are composed of 3–4 subunits: the pore-forming α1 subunit and auxiliary β as well as the α2δ and γ subunits (Hofmann et al., 1994). In the mammalian brain, Ca_v_1.2 and Ca_v_1.3 are the two major α1 subunits of L-VGCCs that constitute an important route of Ca^2+^ entry into neurons.

Here, we attempted to more tightly link the observed deficiency of LTP in TNC^−/−^ mice to a reduced function of L-type calcium channels and to behavioral deficits. We show that in TNC^−/−^ mice, the level of expression of the two L-VGCC α1 subunits is not decreased, but the influx of Ca^2+^ via L-VGCCs is significantly reduced. We further show L-VGCC-dependent impairment in extinction of contextual fear memories in TNC^−/−^ mice. We conclude that impaired functionality of L-VGCCs may be the reason for impairment of LTP and behavioral deficits in TNC^−/−^ mice.

## Materials and Methods

### Mice

TNC^−/−^ mice (Evers et al., 2002) were inbred on the C57BL/6 background. Ten- to twelve-week-old male TNC^−/−^ and TNC^+/+^ littermates were obtained from heterozygous breeding and were kept under an inverted 12:12 h light:dark cycle (lights off at 07:00) and standard housing conditions (23 ± 1°C; 50% humidity; food and water *ad libitum*). Behavioral tests were performed in an experimental room adjacent to the animal facility and illuminated with dim red light. Experiments were performed in the middle of the dark phase of the cycle. All materials were cleaned with soapy water, water and ethanol (75%) between mice. Experiments were carried out in accordance with the European Community Council Directive (86/609/EEC), and the procedures used were approved by the State of Hamburg. Care was taken to minimize pain or discomfort for the animals.

### Analysis of Ca_v_1.2 and Ca_v_1.3 Expression

The polyclonal antibodies against the Ca_v_1.2 and Ca_v_1.3 subunits of L-VGCCs were kindly provided by R. Westenbroek and W. Catterall and are described elsewhere (Hell et al., 1996). Monoclonal antibody against rabbit glyceraldehyde-3-phosphate dehydrogenase (GAPDH) was obtained from Chemicon International (Temecula, CA, USA).

The immunohistochemical analysis of Ca_v_1.2 and Ca_v_1.3 expression was performed as described by Kochlamazashvili et al. (2010). For Western blotting, hippocampi were homogenized in 200 μl TE buffer (50 mM Tris/HCl, 5 mM EDTA, pH 8). After determination of the protein concentration using the BCA^TM^ Protein assay (Thermo Scientific, Rockford, IL, USA), 50 μg proteins per lane were subjected to SDS-PAGE on 10% gels followed by Western blot analysis. Proteins were transferred to a nitrocellulose membrane (Protran, Schleicher and Schuell, Dassel, Germany), and the membrane was blocked with 5% non-fat dry milk powder in PBS, pH 7.5. The membrane was incubated with primary antibody (1:1000) overnight at 4°C with shaking, washed in PBS with 0.05% Tween (PBS-T), and probed with HRP-conjugated secondary antibody (1:10000 in PBS containing 5% milk powder) for 1 h. After washing, immunodetection was performed using the chemiluminescent substrate with extended duration (Pierce, Bonn, Germany) on X-ray films (Kodak Biomax-ML, Sigma-Aldrich). Band intensities were densitometrically quantified using the image software TINA 2.09 (DesignSoft Inc., Budapest, Hungary).

### Recordings of LTP in Hippocampal Slices

After brief CO_2_ sedation, decapitation and removal of the brain, transverse hippocampal sections were cut with a Leica VT 1000 M vibratome (Leica, Nussloch, Germany) in ice-cold artificial cerebrospinal fluid (ACSF) containing (in mM): 250 sucrose, 25 NaHCO_3_, 25 glucose, 2.5 KCl, 1.25 NaH_2_PO_4_, 2 CaCl_2_, and 1 MgCl_2_ (pH 7.3, adjusted with NaOH), as described (Evers et al., 2002). The slices were then kept at room temperature for at least 2 h before the start of recordings in carbogen-bubbled ACSF, containing 125 mM NaCl, instead of 250 mM sucrose. Recordings of field excitatory postsynaptic potential (fEPSP) were performed in the* stratum radiatum* of the CA1b subfield with glass pipettes filled with ACSF and having a resistance of 1–2 MΩ. Schaffer collaterals/commissural fibers were stimulated with a bipolar electrode placed approximately 300 μm closer to the CA3 subfield than the recording electrode. Basal synaptic transmission was monitored at 0.05 Hz. Four TBSs were applied to induce LTP with the inter-TBS interval of 20 s. TBS consisted of 10 bursts delivered at 5 Hz. Each burst consisted of four pulses delivered at 100 Hz. The duration of pulses was 0.2 ms, and the stimulation strength was set to provide baseline fEPSPs with amplitudes of approximately 50% from the subthreshold maximum. To restore LTP in TNC^−/−^ mice, an activator of L-VGCC was added to ACSF for 20 min, starting 15 min before induction of LTP ((+)Bay K-8644, Tocris, Bristol, UK; stock of 100 mM in ethanol, applied at a final concentration of 10 μM).

### Ca^2+^ Imaging

Hippocampal slices (350 μm) of 4- to 6-week-old mice were prepared as described previously (Balaban et al., 2004) and investigated under submerged conditions at 32–34°C. Perfusion medium contained (in mM) 125 NaCl, 2.5 KCl, 2 CaCl_2_, 1.25 NaH_2_PO_4_, 25 NaHCO_3_, 1.5 MgCl_2_, 25 D-glucose and 0.5 L-glutamine and was bubbled with 95% O_2_ and 5% CO_2_. Recordings from pyramidal cells in the CA1 region of the hippocampus were conducted with patch electrodes, containing (in mM) 127 K-gluconate, 20 KCl, 2 MgCl_2_, 2 Na_2_ATP, 10 HEPES and 0.025 of the fluorescent calcium sensitive dye Oregon Green 488 BAPTA 1 (Molecular Probes, Eugene, OR, USA). To induce Ca^2+^ influx, we applied either depolarizing pulses through the recording pipette, or synaptic stimuli through extracellular electrodes. Synaptic stimuli were applied in four theta bursts at 5 Hz. The strength of the synaptic stimuli was adjusted to evoke 1–4 action potentials in each burst. Depolarizing pulses were applied via the recording pipette in a theta-burst-like manner as five pulses at 50 Hz; the duration of each pulse was 10 ms. The strength of the injected current was adjusted so that 3–5 action potentials were evoked by a burst. The recording of Ca^2+^ fluorescence started 20–30 min after rupturing the membrane to let the dye penetrate the cell. For imaging, a CCD camera SenSys1400 (Photometrics, Muenchen, Germany) was used. Acquisition of the imaging data and its synchronization to intracellular stimulation and recording of electrophysiological data was carried out using MetaMorph software (Universal Imaging Corporation, Downingtown, PA, USA). Fluorescence changes of Oregon Green were measured with single wavelength excitation (470 ± 20 nm) and emission >510 nm. Ca^2+^ concentration changes were expressed as ΔF/*F*_0_, where *F*_0_ is the fluorescence intensity when the cell is at rest, and ΔF is the change in fluorescence during stimulation. The signals were normalized per number of action potentials. Ca^2+^ imaging experiments and analyses were performed from two groups of mice without knowledge of their genotypes.

### Contextual Fear Conditioning

This paradigm was used to test long-term memory and extinction of the conditioned response. Mice were conditioned in the context, which consisted of a chamber (23.5 × 23.5 cm and 19.5 cm high) with Plexiglas walls and ceiling, and a stainless-steel grid-floor from which an electric shock could be elicited. The chamber was surrounded by a black curtain and illuminated by white light (10 lux). The test was performed over three consecutive days. On day 1, mice were placed in the center of the cage and received three electric footshocks (0.25 mA, 1 s) at 120, 160, and 200 s. At 240 s the recording was terminated, and mice were immediately returned into their home cage. On day 2, 24 h after conditioning, mice underwent an extinction trial and were placed again in the context for 28 min without receiving any footshock. On day 3, mice underwent a recall trial that consisted of being placed in the conditioning chamber for 4 min without receiving any footshock. The conditioned response was analyzed by quantifying the percentage of time spent freezing (defined as absence of body movements for at least 1 s). Freezing behavior was automatically analyzed using a modified version of the infrared sensor Mouse-E-Motion (Infra-e-motion, Hamburg, Germany). Long-term memory for the context was evaluated by quantification of the amount of time spent freezing during the first 4 min of the extinction trial on day 2. Short- and long-term extinction of the conditioned responses was evaluated by quantification of time spent freezing during the last 4 min of the extinction trial on day 2 and during the recall trial on day 3.

### Drugs for Behavioral Experiments

Nifedipine (25 mg/kg) was suspended in 10% Cremophor EL/PBS vehicle and diltiazem (15 mg/kg) was suspended in saline (0.9% NaCl in water). Intraperitoneal injections were performed 50 min (nifedipine) or 20 min (diltiazem) prior to the recall trial and extinction protocol performed on day 2 of the contextual fear conditioning test.

### Statistical Analysis

Comparisons between two groups were performed with the two-tailed *t*-test. Behavioral data were evaluated using a multifactorial analysis of variance (ANOVA) followed by Newman-Keuls *post hoc* tests when appropriate: two-way ANOVA (with “genotype” and “treatment” as between groups factors), mixed two-way (with “genotype” as between groups factor and “time” as within group factor) and mixed three-way ANOVA (with “genotype” and “treatment” as between groups factors and “time” as within group factor). All tests were two tailed, and the level of significance was set at *p* < 0.05. Data are presented as the mean ± standard error of the mean (SEM).

## Results

### Impaired LTP in TNC^−/−^ Mice Is Rescued by Transient Activation of L-VGCCs during LTP Induction

**Figure 1:**
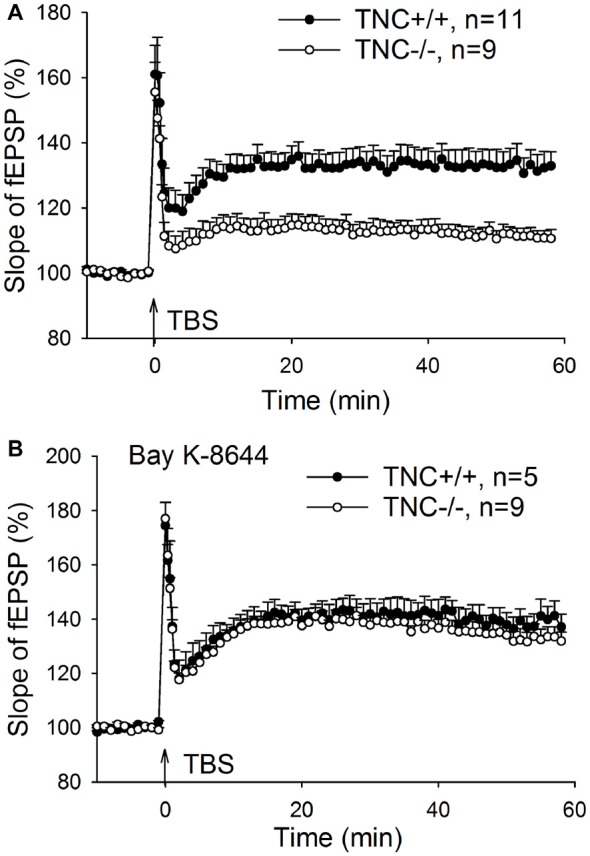
Restoration of CA1 long-term potentiation (LTP) in tenascin-C (TNC^−/−^) mice by the L-type voltage-gated Ca^2+^ channel (L-VGCC) activator Bay K-8644. **(A)** Four trains of theta-burst stimulation (TBS) of Schaffer collateral/commissural fibers induced impaired LTP in the CA1 region of TNC^−/−^ mice. **(B)** LTP in TNC^−/−^ mice can be restored to wild-type levels by the L-VGCC activator Bay K-8644 (10 μM). Mean and SEM values are shown, the mean slope of field excitatory postsynaptic potentials (fEPSPs) recorded 10 min before the induction of LTP was set to 100%.

Our previous work (Evers et al., 2002) demonstrated impairment of LTP in the CA1 area of the hippocampus induced by TBS of Schaffer collaterals/commissural fibers in TNC^−/−^ mice. Here, we first aimed at reproducing these findings. LTP was induced by four episodes (one per 20 s) of TBS consisting of 10 bursts (one per 200 ms) of four pulses at 100 Hz. Figure 1A shows impairment of LTP in TNC^−/−^ mice. The magnitude of LTP in TNC^−/−^ mice (111.3 ± 2.4%, *n* = 11 slices) was significantly reduced compared to LTP in wild-type (TNC^+/+^) mice (132.6 ± 4.8%, *n* = 9, *p* = 0.002). Since our prior work showed that this reduction in LTP magnitude in TNC^−/−^ mice was mimicked by treating slices from TNC^+/+^ mice with the L-VGCC blocker nifedipine and that the effects of TNC deficiency and nifedipine showed full occlusion, we hypothesized that the function of L-VGCCs was impaired in TNC^−/−^ mice (Evers et al., 2002). This hypothesis predicts that impaired LTP in TNC^−/−^ mice might be restored by the upregulation of L-type channel activity. To test this assumption, we used BAY K-8644, an activator of L-VGCCs. Induction of LTP in TNC^−/−^ mice in the presence of BAY K-8644 (bath-applied for 20 min starting 15 min before induction of LTP) restored LTP magnitude (133.2 ± 3.6%, *n* = 9) to the levels seen in TNC^+/+^ mice (132.6 ± 4.8%, *n* = 9, *p* = 0.922; Figure 1B). In TNC^+/+^ mice, the level of LTP induced in the presence of BAY K-8644 (138.4 ± 2.9%, *n* = 5) was not different from the untreated control (*p* = 0.323; Figure 1B). These data support the idea that TNC^−/−^ mice have a partial deficit in the activity of L-VGCCs, which can be compensated by pharmacological activation of these channels. Next, we tested whether reduced activity of L-VGCCs is due to reduced expression or impaired functionality of L-VGCCs.

### L-VGCC Channel Expression Is Not Reduced in Hippocampi of TNC^−/−^ Mice

**Figure 2:**
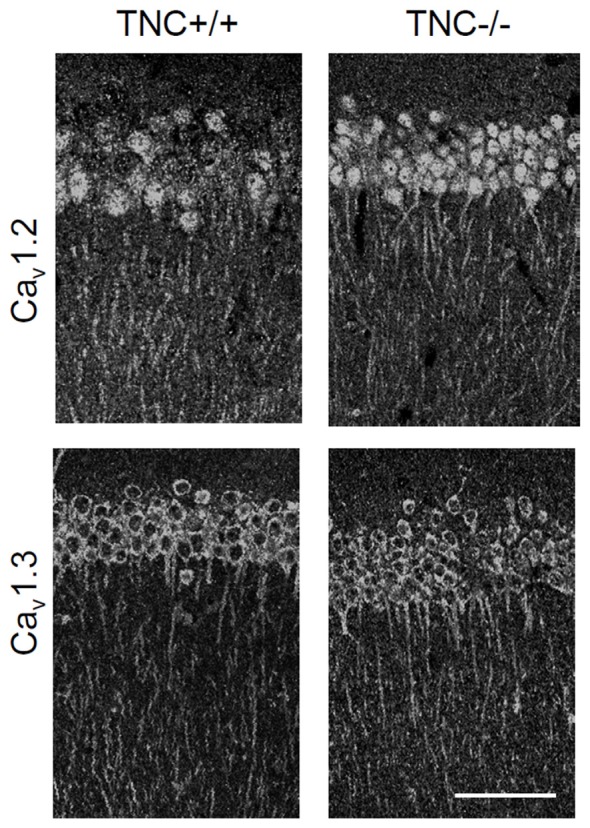
Immunoreactivity for Ca_v_1.2 and Ca_v_1.3 subunits of neuronal L-VGCCs in the CA1 areas of TNC^+/+^ and TNC^−/−^ mice. The strongest signal was observed in somata and apical dendrites of cells located in the *stratum pyramidale*. There was no obvious difference in expression between genotypes, although a slight increase in the expression of Ca_v_1.3 in TNC^−/−^ mice was noticed by comparing images from three pairs of wild-type and knockout mice. Scale bar: 200 μm, applicable to all panels.

We first performed immunohistochemical analysis to estimate whether TNC deficiency affects the overall expression or localization of Ca_v_1.2 and Ca_v_1.3 α1 subunits of neural L-VGCCs. No reduction in expression of either channel subunit was detected in the CA1 area of the hippocampus (Figure 2). Consistent with a previous study (Hell et al., 1996), we observed the strongest expression of L-VGCCs in the somata and proximal dendrites of CA1 pyramidal neurons, but these channels were also apparent in the distal part of dendritic trees.

**Figure 3:**
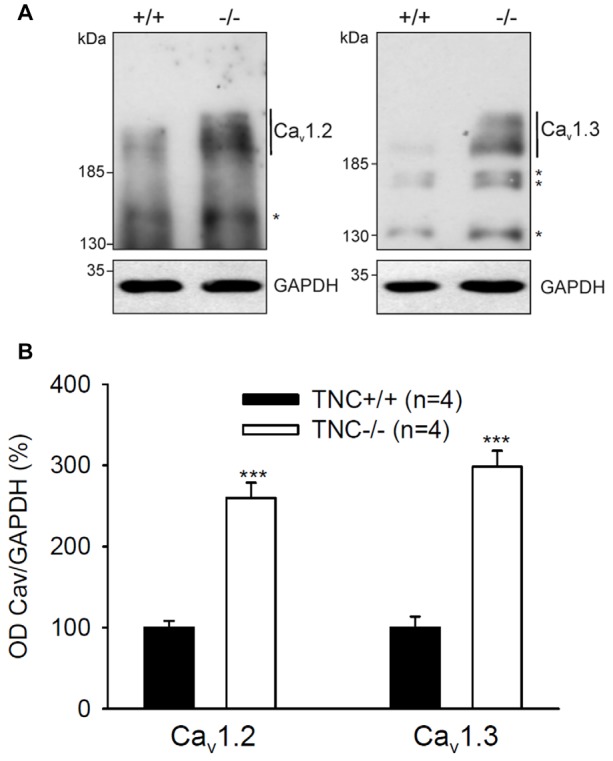
Elevated expression of L-VGCCs in hippocampus of TNC^−/−^ mice. Homogenates of hippocampi from TNC^−/−^ and TNC^+/+^ littermates were subjected to Western blot analysis using antibodies against the Ca_v_1.2 and Ca_v_1.3 subunits of L-VGCCs and against glyceraldehyde-3-phosphate dehydrogenase (GAPDH) to control loading. **(A)** Representative Western blots are shown. **(B)** The amounts of Ca_v_1.2 and Ca_v_1.3 ranging from ~190 kDa to ~220 kDa (indicated by vertical lines) were determined by densitometry and normalized to GAPDH levels. The asterisks in **(A)** indicate bands of unknown identity. Means and SEMs of the expression levels (4 animals for each genotype) were related to the mean value measured for TNC^+/+^ hippocampi, which was set to 100%, ***p < 0.001, *t*-test.

Next, we performed semiquantitative Western blot analysis with equal protein amounts of total homogenate prepared from the hippocampi of TNC^−/−^ and TNC^+/+^ mice. We used affinity purified polyclonal antibodies against the Ca_v_1.2 and Ca_v_1.3 subunits of neural L-VGCCs, which recognized two bands of 210–220 and 180–190 kDa (Hell et al., 1996). The total levels of these two forms were quantified by densitometry and normalized against the GAPDH levels. The levels of GAPDH were nearly identical in the compared probes, showing that it is a proper loading and expression control that can be used for normalization of L-VGCC levels. The results indicated elevated, rather than reduced, expression of these two forms of Ca_v_1.2 and Ca_v_1.3 subunits in the hippocampus of TNC^−/−^ mice compared to TCN^+/+^ mice (Figure 3). It needs to be pointed out that in addition to the bands with the cognate molecular weights for the channel subunits additional bands of unknown identity are seen in Western blots with these antibodies (Figure 2). We therefore decided that these additional bands will not be measured.

In summary, the combined results suggest that there is no deficit in expression of Ca_v_1.2 and Ca_v_1.3 subunits of L-VGCCs in the hippocampus of TNC^−/−^ mice, suggesting that impairment of L-VGCC-dependent LTP mechanisms in TNC^−/−^ mice is due to alteration of either functional properties or cell surface expression of these channels.

### Ca^2+^ Influx via L-VGCCs Is Reduced and Insensitive to Nifedipine in TNC^−/−^ CA1 Pyramidal Neurons

**Figure 4:**
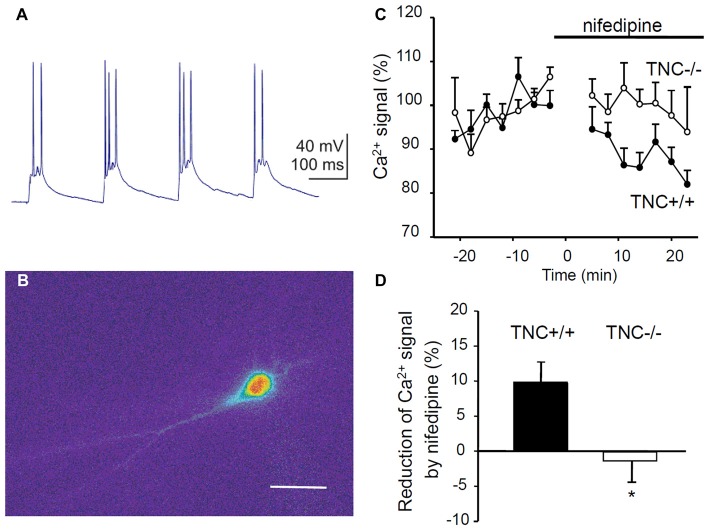
Reduction of Ca^2+^ entry via L-VGCCs in TNC deficient mice. **(A)** Intracellular recording of the CA1 pyramidal cell response to theta-burst synaptic stimulation. In each burst, 2–3 action potentials were generated on depolarization peaks. **(B)** Changes of the fluorescence of the calcium indicator Oregon Green 488 BAPTA-1 during the response of CA1 pyramidal cell to TBS. The panel shows the difference in the fluorescence between two frames acquired with 400 ms exposure before and during the TBS. Scale bar: 40 μm. **(C)** The time-course of Ca^2+^ signal changes induced by application of the L-VGCC blocker nifedipine (horizontal bar). Mean values of Ca^2+^ signals 0–25 min before nifedipine application were set to 100%. **(D)** Summary of the reduction of theta-burst-induced Ca^2+^ signals by nifedipine. Means and SEM of signals measured repeatedly 5–25 min after nifedipine application in 12 TNC^+/+^ and 10 TNC^−/−^ mice are presented, *p < 0.05, *t*-test.

To directly test whether TNC deficiency leads to impaired activity of L-VGCCs, we measured Ca^2+^ influx in TNC^−/−^ and TNC^+/+^ neurons using Ca^2+^ imaging. We filled CA1 pyramidal cells with the Ca^2+^ sensitive dye Oregon Green 488 BAPTA-1 and recorded changes in the fluorescence induced by TBS or trains of depolarization pulses applied via a patch pipette (Figures 4A,B). To assess the contribution of L-VGCCs, we measured Ca^2+^ influx in the control conditions and after the addition of nifedipine to the recording medium. The difference between Ca^2+^ signals recorded under these two conditions characterizes the contribution of L-VGCCs. We revealed a significant difference between nifedipine-sensitive components in TNC^+/+^ and TNC^−/−^ mice. In TNC^+/+^ mice, application of nifedipine led to a clear reduction in the Ca^2+^ signals in the somata of CA1 pyramidal neurons in response to TBS. In TNC^+/+^ mice, the Ca^2+^ signals were reduced by ~10% (to 90.1% of control, reduction by 9.9 ± 3.1%, *n* = 13, *p* = 0.008). In contrast, in TNC^−/−^ mice, the application of nifedipine did not reduce Ca^2+^ signals (101.3% of the control, change of 1.3 ± 3.6%, *n* = 10; *p* = 0.726; Figures 4C,D). The difference between genotypes in the reduction of Ca^2+^ signals by nifedipine (and, thus, in the contribution of L-type channels in the two genotypes) was significant (*p* = 0.022).

To exclude a possible contribution of presynaptic influences, we performed the same measurements but induced Ca^2+^ influx by theta-like bursts of spikes evoked by brief depolarization pulses through the patch pipette at the somata of CA1 pyramidal neurons (five pulses, 10 ms, at 50 Hz, current strength adjusted to evoke 3–5 action potentials per burst). Similar to results with synaptic stimulation, nifedipine significantly reduced Ca^2+^ signals in TNC^+/+^ mice (reduction by 6.2 ± 2.4%, *n* = 13, *p* = 0.024) but not in TNC^−/−^ mice (increase by 2.8 ± 2.7%, *n* = 10, *p* = 0.327). The difference between genotypes in the reduction of Ca^2+^ signals by nifedipine was significant (*p* = 0.017).

Thus, Ca^2+^ entry mediated by L-VGCCs during TBS or theta-like direct postsynaptic activation is impaired in the hippocampus of TNC^−/−^ mice. These results provide strong support for the hypothesis that TNC regulates the activity of L-VGCCs in the CA1 area of the hippocampus (Evers et al., 2002). Next, we asked whether this mechanism plays a role in the regulation of hippocampus-dependent behavior.

### Acquisition and Retention of Long-Term Fear Memory Are Normal, But Extinction Is Impaired in TNC^−/−^ Mice

**Figure 5:**
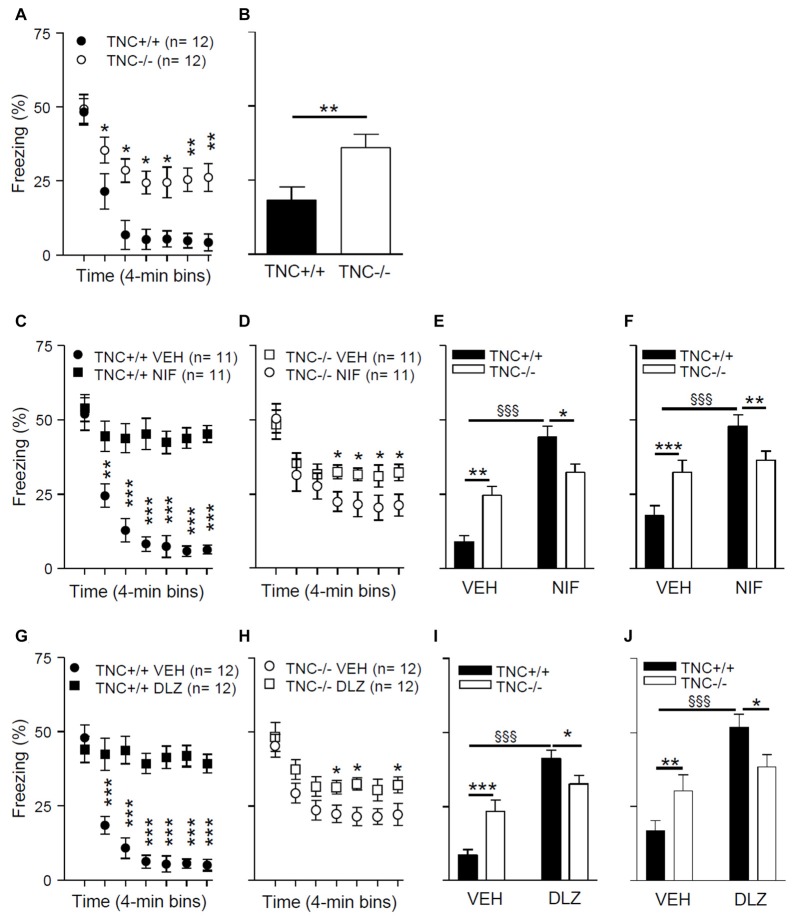
TNC deficiency and pharmacological block of L-VGCCs inhibit the extinction of conditioned fear in mice. **(A)** During the extinction protocol performed on day 2, 24 h after contextual fear conditioning, TNC^−/−^ mice spent more time freezing than TNC^+/+^ mice. *P < 0.05 compared to TNC^+/+^ mice within the same time bin (Newman-Keuls *post hoc* test after mixed two-way analysis of variance (ANOVA)). **(B)** During the 4 min recall trial performed on day 3, TNC^−/−^ mice spent more time freezing than TNC^+/+^ mice. *P < 0.05 compared to TNC^+/+^ mice (*t*-test). **(C−F)** Mice were intraperitoneally (i.p.) injected with either vehicle (VEH) or 25 mg/kg nifedipine (NIF) 20 min before the extinction trial on day 2. **(C)** TNC^+/+^ mice injected with nifedipine (TNC^+/+^ NIF) spent more time freezing starting from the 2nd time bin compared to TNC^+/+^ mice injected with vehicle (TNC^+/+^ VEH). **,***P < 0.01 and 0.001, respectively, compared to TNC^+/+^ NIF mice within the same time bin (Newman-Keuls test after three-way mixed ANOVA). **(D)** TNC^−/−^ NIF mice showed more freezing than TNC^−/−^ VEH mice starting from the 4th time bin. *P < 0.05 compared to TNC^−/−^ NIF mice within the same time bin (Newman-Keuls test after three-way mixed ANOVA). **(E)** Time spent freezing from the 2nd to the last time bin of the extinction trial on day 2. **(F)** Time spent freezing during the 4 min recall trial on day 3. **(G–J)** Mice were i.p. injected with either VEH or 15 mg/kg diltiazem (DLZ) 20 min before undergoing the extinction trial on day 2. **(G)** TNC^+/+^ mice injected with diltiazem (TNC^+/+^ DLZ) spent more time freezing starting from the 2nd time bin compared to TNC^+/+^ mice injected with vehicle (TNC^+/+^ VEH). ***P < 0.001 compared to TNC^+/+^ DLZ mice within the same time bin (Newman-Keuls test after three-way mixed ANOVA). **(H)** TNC^−/−^ DLZ mice showed more freezing than TNC^−/−^ VEH mice during the 4th, 5th and 7th time bins. *P < 0.05 compared to TNC^−/−^ DLZ mice within the same time bin (Newman-Keuls test after three-way mixed ANOVA). **(I)** Time spent freezing from the 2nd to the last time bin of the extinction trial on day 2. **(J)** Time spent freezing during the 4 min recall trial on day 3. In **(E,F,I,J)** *,**,***p < 0.05, 0.01, 0.001, respectively, between TNC^+/+^ and TNC^−/−^ within the same treatment group. ^§§§^*P* < 0.001 between TNC^+/+^ mice of two treatment groups (Newman-Keuls test after two-way ANOVA). Data are shown as means ± SEMs.

To test the possible behavioral consequences of impaired L-VGCC-dependent LTP in TNC^−/−^ mice, we used a paradigm of contextual fear conditioning and extinction, a cognitive function that requires synaptic plasticity in the hippocampus (for review see Izquierdo et al., 2016). On experimental day 1, mice underwent a conditioning protocol to associate a conditioned context (CC) to an unconditional stimulus (a footshock; US). Mice were placed in a test chamber for 4 min, and three series of electric shock pulses (0.25 mA for 1 s) were delivered at 120 s, 160 s and 200 s. Twenty-four hours after conditioning (day 2), we analyzed the behavior during an extinction trial. Mice were re-exposed for 28 min to the CC (same test chamber) in the absence of the US, and the time spent freezing during the extinction trial was measured. Figure 5A shows the time spent freezing during seven 4-min intervals of the extinction trial. The mixed two-way ANOVA (with “genotype” and “time” as between and within groups factors, respectively) showed a significant effect of the interaction between “genotype” and “time” on time spent freezing during the extinction trial (*F*_6,132_ = 10.0; *p* < 0.001). *Post hoc* analyses indicated that there was no difference between TNC^+/+^ and TNC^−/−^ mice during the first 4 min interval (48.3 ± 4.4%, *n* = 12 vs. 49.3 ± 4.9%, *n* = 12, *p* = 0.881). TNC^−/−^ mice spent more time freezing compared to TNC^+/+^ mice during the later phases of the extinction trial (Figure 5A). Thus, whereas long-term memory was intact (as shown by the unaltered freezing time during the first 4 min), short-term extinction was impaired in TNC^−/−^ mice. To test the longer lasting effects of the extinction, we measured time spent freezing 24 h after the extinction trial. On day 3, mice were placed in the CC for 4 min, and TNC^−/−^ mice spent more time freezing compared to TNC^+/+^ animals, indicating that the difference between genotypes acquired during the extinction session was preserved (Figure 5B; *p* = 0.008).

### Reduced Effects of L-VGCC Blockers on Behavior in TNC^−/−^ Mice

In TNC^−/−^ mice, the L-VGCC blocker nifedipine neither affects Ca^2+^ influx into CA1 pyramidal neurons (Figure 4) nor does it reduce the size of LTP (Evers et al., 2002). We thus asked whether the behavioral effects of nifedipine would also be impaired in TNC^−/−^ mice. Pharmacological blockade of L-VGCCs is known to inhibit the extinction of fear (Cain et al., 2002; Izquierdo et al., 2016), although it remains a matter of debate whether these effects are due to impairment of specific cognitive processes mediating extinction or caused by a more generalized motor inhibition (Waltereit et al., 2008; Singewald et al., 2015). We performed contextual fear conditioning and extinction tests as described above using TNC^+/+^ and TNC^−/−^ mice injected with either vehicle or the L-type VGCC blockers nifedipine or diltiazem 20 min before the extinction trial on day 2. Diltiazem was used to confirm that possible effects induced by the pharmacological blockade of the L-type VGCCs are not caused by side effects already described for nifedipine (Waltereit et al., 2008).

The results of these experiments (Figures 5C–E,G–I) revealed a significant effect of the interaction between “genotype”, “treatment” and “time” on time spent freezing (nifedipine experiment: *F*_(6,240)_ = 10.29; *p* < 0.0001; diltiazem experiment: *F*_(6,264)_ = 19.4; *p* < 0.0001; mixed three-way ANOVA with “genotype” and “treatment” as between groups factors and “time” as a within groups factor). The results of *post hoc* analyses indicated that L-VGCC blockers enhance the time spent freezing after 5 min of the extinction trial on day 2 in TNC^+/+^ mice compared to vehicle treated TNC^+/+^ mice (Figures 5C,G). The L-VGCC blockers had similar effects, although less prominently, in TNC^−/−^ mice: time spent freezing was enhanced after 13 min in nifedipine- (Figure 5E) and diltiazem-treated mice (Figure 5H) compared to TNC^−/−^ mice injected with the vehicle. Because the extinction of the conditioned response starts after approximately 5 min of the extinction trial, we performed a two-way ANOVA evaluation on time spent freezing from 5 min to the end of the extinction trial and detected a significant effect of the interaction between “genotype” and “treatment” (*F*_(1,44)_ = 14.05; *p* = 0.0005). *Post hoc* analyses indicated that nifedipine (Figure 5E) and diltiazem (Figure 5I) significantly enhanced the time spent freezing by TNC^+/+^ mice, whereas in the TNC^−/−^ group values obtained with blockers did not reach a statistically significant difference (nifedipine: *p* = 0.061; diltiazem: *p* = 0.087). Moreover, in the vehicle group TNC^−/−^ mice spent more time freezing than TNC^+/+^ mice, but less time than TNC^+/+^ mice in the nifedipine (Figure 5E) and diltiazem (Figure 5I) groups. Similar effects of the interaction between “genotype” and “treatment” were observed on time spent freezing during the 4 min trial on day 3, 24 h after the extinction trial (Figure 5F for nifedipine: *F*_(1,40)_ = 10.1; *p* = 0.0029; Figure 5J for diltiazem: *F*_(1,44)_ = 7.4; *p* = 0.0093).

In conclusion, the inhibitory effects of L-VGCCs blockers on extinction were less pronounced in TNC^−/−^ than in TNC^+/+^ mice, indicating an occlusion of the involved mechanisms.

## Discussion

In the present study, we have shown that impaired L-VGCC-dependent LTP in TNC^−/−^ hippocampi is not caused by reduced expression of L-VGCC α1 subunits, but it is most likely due to a reduced influx of Ca^2+^ via L-type channels. Moreover, our behavioral data suggest that reduced activity of L-VGCCs may underlie the impaired extinction of conditioned fear observed in TNC deficient mice.

Our Ca^2+^ imaging data directly show, for the first time, that L-VGCC-dependent Ca^2+^ transients elicited by TBS of the CA3 input to CA1 pyramidal cells are impaired in TNC^−/−^ mice. As there is no reduction in the expression of the major Ca_v_1.2 and Ca_v_1.3 subunits of L-VGCCs and the LTP-promoting function of channels can be rescued by acute application of the L-channel activator Bay K-8644, we conclude that in the constitutive absence of TNC, L-VGCCs are expressed but are less active than in the presence of TNC.

Similarly, we previously observed that enzymatic removal of other major ECM components, specifically hyaluronic acid and heparan sulfates, results in the impairment of L-VGCC-dependent forms of LTP, which could be rescued by Bay K-8644 (Kochlamazashvili et al., 2010; Minge et al., 2017). Hyaluronic acid was found to acutely facilitate activation of L-VGCCs in a heterologous expression system (Kochlamazashvili et al., 2010), but no direct binding of hyaluronic acid to peptides representing the extracellular domains of Ca_v_1.2 could be detected (Garau et al., 2015). Heparan sulfates can bind to the extracellular domains of Ca_v_1.2 and shorten voltage-dependent inactivation of L-VGCCs containing this subunit in response to prolonged depolarizing pulses in a heterologous system, but failed to modulate Ca^2+^ influx into CA1 pyramidal neurons during postsynaptic depolarization mimicking theta-burst afferent stimulation (Garau et al., 2015). The removal of heparan sulfates was found to affect organization of the distal part of the axonal initial segment and excitability, which in turn reduced the number of action potentials and hence Ca^2+^ influx through L-VGCCs (Minge et al., 2017). Our present demonstration of impaired Ca^2+^ influx in TNC^−/−^ mice suggests that either L-VGCCs are not fully functional in the absence of TNC or that depolarization is not sufficient enough to activate them. Since the excitatory synaptic input to CA1 pyramidal cells is normal in TNC^−/−^ mice (Evers et al., 2002) and reduction in Ca^2+^ influx (normalized per number of generated spikes) was observed in response to theta-like direct depolarization of postsynaptic cells, we conclude that the first assumption is more plausible, i.e., that TNC supports the activity of L-VGCCs.

Since it is known that TNC binds to heparin and heparan sulfate proteoglycans, namely, syndecans and glypicans, as well as to chondroitin sulfate proteoglycans neurocan and phosphacan (Jones and Jones, 2000), which form a complex with hyaluronic acid, it is possible that TNC may potentiate L-VGCC activities via these molecules. Additionally, TNC may interact with diverse integrins, including the fibronectin receptors, namely, α5β1 integrins (Jones and Jones, 2000; Tanaka et al., 2014; Jachetti et al., 2015). Activation of these integrins with beads coated with fibronectin or α5 integrin antibodies in arteriolar smooth muscles potentiates L-VGCC currents through a tyrosine phosphorylation cascade involving Src family tyrosine kinases and various focal adhesion proteins (Wu et al., 1998, 2001). The relevance of this mechanism to TNC-mediated regulation of neural L-VGCCs is supported by our analysis of the effects mediated by a fragment of TNC containing the fibronectin type-III repeats 6–8 (FN6–8), which represents a TNC-fibronectin interaction site that is important for the integration of TNC in the fibronectin-based ECM (Chung et al., 1995). This fragment binds to pyramidal cell somata in the hippocampal formation and repels axons of pyramidal neurons when presented as a border *in vitro* (Strekalova et al., 2002). Injection of this FN6–8 fragment into the hippocampus, which may disrupt the interaction of TNC with fibronectin and other extracellular matrix partners, reduced LTP in the CA1 region (Strekalova et al., 2002).

We have previously shown that cognitive functions are not affected in TNC^−/−^ mice when tested for short- and long-term memory in the fear conditioning, step-down and water maze paradigms (Evers et al., 2002; Morellini and Schachner, 2006). Since pharmacological blockade of L-VGCCs is known to inhibit extinction of fear memories (Singewald et al., 2015), we hypothesized that TNC^−/−^ mice do not properly extinguish conditioned responses when tested in the contextual fear conditioning paradigm. Our data demonstrate that extinction is indeed impaired in TNC^−/−^ mice compared to TNC^+/+^ littermates. The impaired extinction of TNC^−/−^ mice does not appear to be due to general hypolocomotion or reduced exploration because several studies have shown that constitutive TNC deficiency leads to hyperlocomotion and enhanced novelty-induced exploration (Kiernan et al., 1999; Morellini and Schachner, 2006; Stamenkovic et al., 2017). Thus, it is very likely that the inability of TNC^−/−^ mice to inhibit freezing behavior after prolonged re-exposure to the CC reflects cognitive deficits, namely, impaired extinction of context-related conditioned fear responses. Inspired by the effect of Bay K-8644 in LTP experiments, we also attempted to rescue extinction by local injection of Bay K-8644 in TNC^−/−^ and TNC^+/+^ mice. However, this drug caused drastically reduced motor activity, Straub tail reaction and muscular clonus and tonus, precluding proper analysis of cognitive functions.

It is noteworthy that extinction of conditioned fear could only be partially inhibited by nifedipine and diltiazem in TNC^−/−^ mice, whereas it was completely abolished by these L-VGCC blockers in TNC^+/+^ mice. This observation suggests that other mechanisms independent of L-VGCCs are responsible for the residual extinction in TNC^−/−^ mice. Such mechanisms are likely to be activated as a compensatory response to the reduced activity of the L-type channels in the absence of TNC. Indeed, we observed that expression of the Ca_v_1.2 and Ca_v_1.3 subunits was enhanced in TNC^−/−^ vs. TN^+/+^ hippocampi, suggesting that compensatory mechanisms occur to support proper cellular function and adaptive behavior in TNC^−/−^ mice.

Our data support the view that the impaired fear extinction in TNC^−/−^ mice is due to reduced function of L-VGCCs. Whereas the inhibitory effects of Ca_v_1.2 and Ca_v_1.3 blockers on the extinction of fear memories are well documented (Cain et al., 2002; Striessnig et al., 2006) and support the view that these channels plays a role in fear extinction, the deleterious effects of L-VGCC blockers on fear extinction could be due to a peripheral action of the drugs, challenging the idea that L-VGCCs play a direct role in the cellular processes controlling extinction (Waltereit et al., 2008). However, local injection of nifedipine or verapamil in the basolateral amygdala (Davis and Bauer, 2012) or hippocampus (de Carvalho Myskiw et al., 2014) also impaired the consolidation of fear extinction, supporting the idea that L-VGCCs play a role in synaptic events mediating extinction in these brain regions. Thus, it is tempting to speculate that the impaired fear extinction in TNC^−/−^ mice is due to the reduced function of L-VGCCs in the hippocampus or basolateral amygdala. On the other hand, we cannot exclude the possibility that TNC deficiency affects L-VGCC function also in other brain regions controlling fear responses, such as the prefrontal or perirhinal cortices (Kent and Brown, 2012; Izquierdo et al., 2016). It is likely that other behavioral impairments of TNC^−/−^ mice are also caused by reduced function of L-VGCCs. It is noteworthy in this respect that mice deficient for Ca_v_1.3 show behavioral alterations similar to those observed in TNC^−/−^ mice (Morellini and Schachner, 2006), such as reduced novelty-induced anxiety and reduced use of passive behavioral coping strategies (Busquet et al., 2010). Additionally, mice deficient in the Ca_v_1.2 channel exhibit a reduction in their ability to phase-advance circadian behavior when subjected to a light pulse at late night (Schmutz et al., 2014), which is consistent with the delayed resynchronization of circadian activity observed in TNC^−/−^ mice (Morellini and Schachner, 2006). In conclusion, the present data suggest a functional link between TNC and L-VGCCs that may be relevant for several brain regions and different behaviors.

Interestingly, in patients with Alzheimer’s disease (AD), TNC is co-expressed in Aβ plaques (Mi et al., 2016). Additionally, in a mouse model of AD, TNC expression is upregulated, while TNC deficiency reduces pro- but enhances anti-inflammatory functions in the AD model and is associated with a reduced cerebral Aβ load and higher levels of the postsynaptic density protein 95 (Xie et al., 2013). Considering the role of TNC in the regulation of L-VGCCs (present data) and long-term depression (Evers et al., 2002), it is plausible to assume that a reduction in L-VGCC-dependent depression may overlap with the anti-inflammatory effects of TNC deficiency and contribute to the preservation of the postsynaptic machinery in the AD model. Moreover, genetic variability in L-VGCCs has been found to have pleiotropic effects on psychopathology associated with autism spectrum disorder, attention deficit-hyperactivity disorder, bipolar disorder, major depressive disorder, and schizophrenia (Cross-Disorder Group of the Psychiatric Genomics Consortium, 2013). Because TNC appears to be a major regulator of L-VGCC activity, it is highly tempting to speculate that TNC is linked to some of these disorders.

## Author Contributions

FM, MV, RK, MS and AD designed the experiment. FM, AM, MV, MC, GP, LF and RK collected and analyzed the data. FM, MV, MS and AD wrote and revised the manuscript; all authors approved the final version of the manuscript.

## Conflict of Interest Statement

The authors declare that the research was conducted in the absence of any commercial or financial relationships that could be construed as a potential conflict of interest.
